# Detection of Ligation Products of DNA Linkers with 5′-OH Ends by Denaturing PAGE Silver Stain

**DOI:** 10.1371/journal.pone.0039251

**Published:** 2012-06-27

**Authors:** Feng Gao, Huafu Zhou, Wei Li, Xuerong Zhang

**Affiliations:** 1 Department of Anal and Colorectal Surgery, The First Affiliated Hospital of Guangxi Medical University, Nanning, Guangxi, China; 2 Department of Cardiothoracic Surgery, The First Affiliated Hospital of Guangxi Medical University, Nanning, Guangxi, China; 3 Medical Scientific Research Center, Guangxi Medical University, Nanning, Guangxi, China; Temple University, United States of America

## Abstract

To explore if DNA linkers with 5′-hydroxyl (OH) ends could be joined by commercial T4 and E. coli DNA ligase, these linkers were synthesized by using the solid-phase phosphoramidite method and joined by using commercial T4 and E. coli DNA ligases. The ligation products were detected by using denaturing PAGE silver stain and PCR method. About 0.5–1% of linkers A–B and E–F, and 0.13–0.5% of linkers C–D could be joined by T4 DNA ligases. About 0.25–0.77% of linkers A–B and E–F, and 0.06–0.39% of linkers C–D could be joined by E. coli DNA ligases. A 1-base deletion (-G) and a 5-base deletion (-GGAGC) could be found at the ligation junctions of the linkers. But about 80% of the ligation products purified with a PCR product purification kit did not contain these base deletions, meaning that some linkers had been correctly joined by T4 and E. coli DNA ligases. In addition, about 0.025–0.1% of oligo 11 could be phosphorylated by commercial T4 DNA ligase. The phosphorylation products could be increased when the phosphorylation reaction was extended from 1 hr to 2 hrs. We speculated that perhaps the linkers with 5′-OH ends could be joined by T4 or E. coli DNA ligase in 2 different manners: (i) about 0.025–0.1% of linkers could be phosphorylated by commercial T4 DNA ligase, and then these phosphorylated linkers could be joined to the 3′-OH ends of other linkers; and (ii) the linkers could delete one or more nucleotide(s) at their 5′-ends and thereby generated some 5′-phosphate ends, and then these 5′-phosphate ends could be joined to the 3′-OH ends of other linkers at a low efficiency. Our findings may probably indicate that some DNA nicks with 5′-OH ends can be joined by commercial T4 or E. coli DNA ligase even in the absence of PNK.

## Introduction

Nicks and breaks in the DNA double helix can result from DNA replication or DNA damage. DNA ligases are the critical enzymes participating in the repair of these nicks. A variety of different DNA ligases have been found, such as T4 DNA ligase, E. coli DNA ligase, and DNA ligases I, II, III, and IV. They are classified into two groups based on their cofactors: the ATP-dependent DNA ligases and the NAD^+^-dependent DNA ligases. ATP-dependent DNA ligases are derived from eukaryotic cells, T series bacteriophages and archaebacteria. NAD^+^-dependent DNA ligases were found in eubacteria [1]–[3]. In 1997, it was found that ATP-dependent ligase was also expressed in the respiratory pathogen haemophilus influenzae [4]. In addition, some bacterial species such as Neisseria meningitidis have been found to encode both ATP-dependent ligase homologues and NAD^+^-dependent ligases simultaneously [5]–[7].

Each type of DNA ligase possesses different functions. For example, DNA ligase I is involved in the ligation of Okazaki fragments and some repair pathways, and DNA ligase IV is required for V(D)J recombination [2], [8]. T4 DNA ligase is an ATP-dependent ligase that repairs single-strand nicks in duplex DNA, RNA or DNA/RNA hybrids but has no activity on single-stranded nucleic acids [9]–[10]. E. coli DNA ligase is a NAD^+^-dependent ligase from Escherichia coli, and it works the best on cohesive dsDNA ends and is also active on nicked DNA. In the presence of certain macromolecules such as polyethylene glycol, E. coli DNA ligase can also catalyze the ligation between DNA blunt ends [11].

We found occasionally that PCR fragments generated by using the primers with 5′- OH groups could be joined by T4 DNA ligase when we joined these PCR fragments to form a larger DNA fragment [12]. Because DNA ligases are important for DNA replication and injury repair, and some experiments such as molecular cloning and deep sequencing are involved in the dephosphorylation of DNA linkers, we wanted to explore whether DNA linkers with 5′- OH ends could be joined by commercial T4 and E. coli DNA ligases.

## Methods

### Synthesis of DNA Linkers with 5′- OH Ends

**Figure 1:**
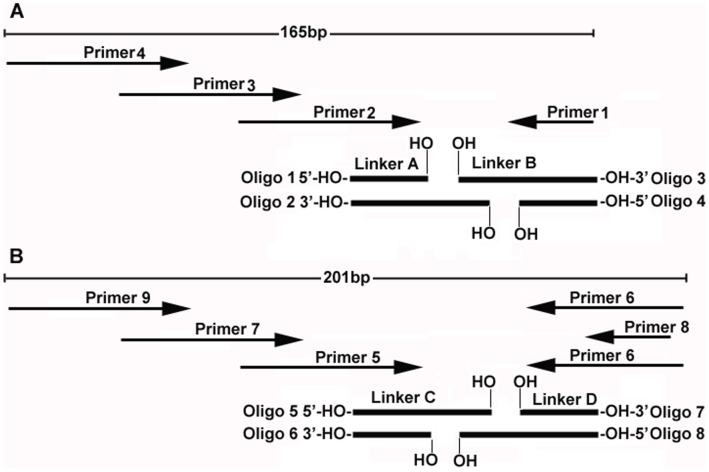
Schematic diagram of linkers A–B, C–D and the three-round overlap PCR primers. The arrows represent PCR primers. The sequences of linkers and PCR primers are listed in Tables 1 and 2, respectively. (**A**) Linkers A–B and PCR primers. (**B**) Linkers C–D and PCR primers.

Two types of DNA linkers with 5′-OH ends were synthesized, without any chemical modifications, by Shanghai Sangon (China) with an ABI 3900 DNA synthesizer and the solid-phase phosphoramidite method. After synthesis, these linkers were purified by using high affinity purification (HAP) or polyacrylamide gel electrophoresis (PAGE) plus high-performance liquid chromatography (HPLC) methods. Their purity was ≥98%. Most of the remaining 2% should be the other oligos with 5′-OH ends because it was impossible that an oligo with 5′-phosphate end could be synthesized with the solid-phase phosphoramidite method. Type I linkers included linkers A, B, E, F, G and H containing protruding 5′-OH ends. Type II linkers included linkers C and D containing recessive 5′-OH ends. Linkers A, C, E, and G are complementary to linkers B, D, F, and H, respectively. Linkers E and F are 58–59 bp long, and the other linkers are 28–30 bp long. Short linkers can be separated from the ligation products more easily than the long ones by using a PCR product purification kit. There is a 1-nucleotide deletion at the 5′-ends of linkers G and H. All of these linkers are showed in Table 1 or Figure 1.

**Table 1:** DNA sequences of the linkers.

Linker type	Linker	Oligo	Sequences (5′-OH→3′-OH)	Length(nt)
I	A	1	TCGGCATGGACAGGAACCGAGA	22
		2	CTCTATTCTCTCGGTTCCTGTCCATGCCGA	30
I	B	3	GAATAGAGACTGACTCTCTCTGCCTATT	28
		4	AATAGGCAGAGAGAGTCAGT	20
II	C	5	CTTAGACCTCACCCTGTGGAGCAAGAGTG	29
		6	CTCCACAGGGTGAGGTCTAAG	21
II	D	7	TGGCAGGTTGGTATCAAGGTT	21
		8	AACCTTGATACCAACCTGCCACACTCTTG	29
I	E	9	GAGGTGTCCCACTCCAGATTCACTACTGTCGGCATGGACAGGAACCGAGA	50
		10	CTCTATTCTCTCGGTTCCTGTCCATGCCGACAGTAGTGAATCTGGAGTGGGACACCTC	58
I	F	11	GAATAGAGACTGACTCTCTCTGCCTATTGGTCTATTTTCCCACCCTTAGGCTGCTGGTG	59
		12	CACCAGCAGCCTAAGGGTGGGAAAATAGACCAATAGGCAGAGAGAGTCAGT	51
I	G	1	TCGGCATGGACAGGAACCGAGA	22
		13	TCTATTCTCTCGGTTCCTGTCCATGCCGA	29
I	H	14	AATAGAGACTGACTCTCTCTGCCTATT	27
		4	AATAGGCAGAGAGAGTCAGT	20

### Ligations between Complementary Linkers

#### Ligations of the linkers with 5′-OH ends

The ligations of linkers A–B, C–D, and E–F by using T4 DNA ligase were performed in 100 µl of T4 DNA ligase reaction mixture containing 1 x T4 DNA ligation buffer (40 mM Tris-HCl, 10 mM MgCl_2_, 10 mM DTT, and 0.5 mM ATP; pH 7.8 at 25°C), 1 µM of each oligo, and 0.25 Weiss units/µl of T4 DNA ligase (Fermentas, Lithuania; Promega, USA; and Takara, Japan). The ligations of linkers A–B, C–D, and E–F by using E. coli DNA ligase were performed in 50 µl of E. coli DNA ligase reaction mixture containing 1 x E. coli DNA ligation buffer (30 mM Tris-HCl, 4 mM MgCl_2_, 10 mM (NH_4_)_2_SO_4_, 1.2 mM EDTA, and 0.1 mM NAD^+^; pH 8.0 at 25°C), 1 x BSA (0.005%), 2 µM of each oligo, and 6 U/µl of E. coli DNA ligase (Takara). To figure out if the ligation of linkers A–B and E–F could be inhibited by (NH_4_)_2_SO_4_, the ligation of these linkers were performed in 100 µl of T4 DNA ligase reaction mixture containing 7.5 mM (NH_4_)_2_SO_4_. The other ligation conditions were the same as above. To see if T4 PNK could use NAD^+^ as its phosphate donor, oligos 2 and 3 of linkers A–B were separately preincubated with T4 PNK in 100 µl of E. coli DNA ligase reaction mixture containing 1 x E. coli DNA ligase buffer, 1 x BSA, 1 µM of each oligo, and 40 U of T4 PNK (Takara). This mixture without ATP was incubated at 37°C for 30 min, and then extracted once with an equal volume of fresh phenol/chloroform (1∶1), and once with chloroform/isopentyl alcohol (24∶1). DNA was precipitated with 3 volumes of 100% ethanol and 1/10th volume of 3 M sodium acetate (pH 5.2) at −20°C for 2 hrs. DNA pellets were washed twice with 300 µl of 75% cold ethanol, dried at room temperature for about 2 hrs, and resuspended in 4 µl of sterilized ddH_2_O. The ligation was then performed in 50 µl of E. coli DNA ligase reaction mixture containing the resuspended oligos 2 and 3 (4 µl each), 2 µM of each of oligos 1 and 4, and the other components mentioned above. All of the ligase reaction mixtures mentioned above and the negative controls without ligase were incubated at 18°C for 10 hrs.

#### Ligation of the linkers phosphorylated by T4 PNK

To compare the ligation products between the linkers with 5′-phosphate ends and the linkers with 5′-OH ends, linkers A–B, C–D and G–H were separately phosphorylated by using T4 PNK. The phosphorylation reactions for linkers A–B, C–D, and G–H were performed in 20 µl, 20 µl and 50 µl of the phosphorylation mixtures, respectively. These phosphorylation mixtures contained 1 x T4 DNA ligation buffer (40 mM Tris-HCl, 10 mM MgCl_2_, 10 mM DTT, and 0.5 mM ATP, pH 7.8 at 25°C), 2 µM of each oligo, and 0.5 U/µl of T4 PNK (Takara). They were incubated at 37°C for 30 min, and then extracted once with phenol/chloroform (1∶1), once with chloroform/isopentyl alcohol (24∶1), precipitated with 3 volumes of 100% ethanol and 1/10th volumes of sodium acetate, washed twice with 100 µl of 75% ethanol, dried at room temperature for 1.5 hrs, resuspended in 8 µl, 8 µl, and 10 µl of ddH_2_O for the oligos of linkers A–B, C-D and G–H, respectively. The ligation of the phosphorylated linkers A–B, C–D, and G–H were performed in 10 µl, 10 µl, and 100 µl of T4 DNA ligase reaction mixtures, respectively. These ligase reaction mixtures contained 1 x T4 DNA ligation buffer, 1 µl of each phosphorylated oligos of linkers A–B and C–D or 10 µl of each phosphorylated oligos of linkers G–H, 1 µM of every other oligo, and 0.28 U/µl of T4 DNA ligase (Takara). The ligation of the phosphorylated linkers A–B and C–D were also performed in 10 µl of E. coli DNA ligase reaction mixtures containing 1 x E. coli DNA ligation buffer, 1 x BSA, 1 µl of each phosphorylated oligos, 1 µM of every other oligo, and 6 U/µl of E. coli DNA ligase (Takara). The ligase reaction mixtures were incubated at 18°C for 10 hrs, and then 65°C for 10 min. 2.5 µl of ligation products of the phosphorylated linkers A–B and C–D were loaded on 15% denature PAGE.

#### Ligation of linkers A–B treated with calf intestine alkaline phosphatase (CIAP)

Based on the principle of the solid-phase phosphoramidite method, we supposed that it should be impossible that the oligos with 5′-phosphate groups could be synthesized by using the solid-phase phosphoramidite method without any chemical modification. Even so, to see if the ligation of linkers A–B could be inhibited by CIAP, oligos 2 and 3 of linkers A and B were separately treated with CIAP before ligation. CIAP treatment of oligos 2 and 3 was performed in 100 µl of CIAP reaction mixture containing 1 x CIAP buffer (0.01 M Tris-HCl, pH 7.5 at 37°C and 0.01 M MgCl_2_), 1 µM of each of oligos 2 and 3, and 0.05 U/µl of CIAP (Fermentas), incubated at 37°C for 30 min, mixed with 1 µl of 0.5 M EDTA (pH 8.0), inactivated at 85°C for 15–90 min, and then extracted twice with an equal volume of phenol/chloroform (1∶1), once with chloroform/isopentyl alcohol (24∶1), and precipitated with ethanol as described above. DNA pellets were resuspended in 10 µl of sterilized ddH_2_O. The resuspended oligos were joined by using T4 and E. coli DNA ligases, respectively. The ligase reaction mixture (50 µl) contained the resuspended oligos 2 and 3 (5 µl each), 1 µM each of oligo 1 and 4, and the other components mentioned above. A positive control without CIAP treatment and a negative control without ligase were included for each ligation reaction. All of the ligase reaction mixtures and the controls were incubated at 37°C for 1 hr, and then at 18°C for 10 hrs.

### Denaturing PAGE Silver Stain

The ligation products of linkers A–B, C–D, E–F, and G–H were 50, 50, 109, and 49 bp long, respectively. These ligation products were extracted with phenol/chloroform before PAGE. Briefly, 50–100 µl of ligation products were extracted once with an equal volume of fresh phenol/chloroform (1∶1), precipitated with ethanol as described above. DNA pellets were washed once with 300 µl of 75% ethanol, dried at room temperature for about 2 hrs, and resuspended in 4 µl, 4 µl, 10 µl, or 8 µl of 1 x TE for the ligation products of linkers A–B, C–D, E–F, or the phosphorylated linkers G–H, respectively. 4 µl of the resuspended ligation products of linkers A–B or C–D were mixed with 2 µl of formamide loading buffer containing 98% (v/v) formamide, 2% (v/v) 0.5 M EDTA, pH 8.0, and 0.1% (w/v) bromophenol blue, denatured at 95°C for 1 min, cooled quickly on ice. 2 µl of the resuspended ligation products of linkers E–F were mixed with 1 µl of 1 x TE and 1 µl of loading buffer containing 40% sucrose and 0.25% (w/v) bromophenol blue. 1 µl of the resuspended ligation products of linkers G–H phosphorylated by T4 PNK was mixed with 2 µl of the resuspended negative control of linkers A–B and 2 µl of formamide loading buffer, denatured at 95°C for 1 min and cooled quickly on ice. These ligation products were then loaded on 12% denaturing PAGE gel (10×10×0.03 cm, A:B  = 19∶1, 7 M urea and 0.5 x TBE) or 15% denaturing PAGE gel (10×10×0.03 cm, A:B  = 29∶1, 7 M urea and 0.5 x TBE). Oligo 15 (5′-GGC AGG TTG GTA TCA AGG TTA CAA GAC AGG TTT AAG GAG ACC AAT AGA AA-3′) was synthesized by Shanghai Sangon and used as a 50-nt DNA marker. Electrophoreses were run in 0.5 x TBE, 25°C, 200 V for 1.7 hrs, or 100 V for 3.5–4.3 hrs. Next, the gels were silver-stained [13]. Briefly, the gels were fixed with 50 ml of 50% methanol containing 10% acetic acid for about 2 hrs, and then washed 4 times (2 min each for the first 2 times, and 5 min each for the last 2 times) with deionized water (200 ml each), stained with 30 ml of 0.1% AgNO_3_ containing 45 µl of 37% formaldehyde for 30 min, developed with 30 ml of 3% NaCO_3_ containing 45 µl of 37% formaldehyde and 30 µl of 2 mg/ml Na_2_S_2_O_3_ until DNA bands appeared clearly, and then stopped the silver stain with 25 ml of 10% acetic acid.

### PCR and DNA Sequencing

The ligation products of linkers A–B and C–D were amplified by three round overlap PCR, and the third round PCR products were analyzed by DNA sequencing. PCR and DNA sequencing primers were synthesized by Shanghai Sangon (Table 2 and Figure 1). PCR was performed in 10–50 µl of PCR mixture containing 1 x PCR buffer (10 mM Tris-HCl, pH 8.0 at 25°C, 50 mM KCl, and 0.08% Nonidet P40), 1.5 mM MgCl_2_, 0.125 mM dNTPs, 0.2 µM of each primer, and 0.1 U/µl of Taq DNA polymerase (Fermentas). 1 µl of PCR template was added for every 10 µl of PCR mixture. The ligation products diluted 1- to 30-fold with sterilized ddH_2_O were used as the first round PCR templates. To improve the background of DNA sequencing, the ligation products (100 µl each) of linkers A–B and C–D were purified by using a PCR product purification kit (GK2051, Generay Biotech, China) following the manufacturer’s instructions. The purified ligation products were then diluted 1- to 30-fold with sterilized ddH_2_O and used as the first round PCR template. The previous round PCR products were diluted 10- to 30-fold with sterilized ddH_2_O and used as the next round PCR template. To characterize the 50-bp DNA band from the ligation products of linkers A–B, the band was cut from the denaturing PAGE gel and used as the first round PCR template. The first and the second round PCRs were performed at 95°C for 3 min, followed by 15 cycles of 94°C for 30 s, and 65°C for 40 s. The third round PCR were carried out at 95°C for 3 min, followed by 33 cycles of 94°C for 30 s, and 65°C for 50 s for Type I linkers or 64°C for 50 s for Type II linkers. 5 µl of PCR products were checked by 2.5% agarose gel electrophoresis with 0.5 µg/ml ethidium bromide (EB). The third round PCR products were sequenced by using a BigDye Terminator v2.0 kit and an automatic sequencer (ABI PRISM 377-96). To analyze the third round PCR products from the negative control of linkers A–B cut from the denaturing PAGE gel, the second round PCR for this negative control was run 25 cycles to generate enough templates for the third round PCR, and then the third round PCR products were sequenced. DNA sequencing data in this research are not new ones that need to be deposited in GenBank.

**Table 2:** Primers used in the three round overlap PCR and DNA sequencing.

Primers	Sequence (5′-OH→3′-OH)
1	AATAGGCAGAGAGAGTCA
2	GAGGTGTCCCACTCCAGATTCACTACTGTCGGCATGGACAGGAACCGAGA
3	TACTTTCCCTAATCTCTTTCTTTCAGGGCAATAATGATACAATGAGGTGTCCCACTCC
4	TTAGTAGCAATTTGTACTGATGGTATGGGGCCAAGAGATATATCTACTTTCCCTAATCT
5	AGAGCCAAGGACAGGTACGGCTGTCATCACTTAGACCTCACCCTGT
6	TTTCTATTGGTCTCCTTAAACCTGTCTTGTAACCTTGATAC
7	AGGGCTGAGGGTTTGAAGTCCAACTCCTAAGCCAGTGCCAGAAGAGCCAAGGACA
8	CTATTGGTCTCCTTAAACCTGTCTTG
9	AGTAGCAATTTGTACTGATGGTATGGGGCCAAGAGATATATCTTAGAGGGAGGGCTGAG
10^a^	AGTAGCAATTTGTACTGATGGTATGG

a DNA sequencing primer.

### Kinase Assay for T4 DNA Ligase

To explore the ligation mechanism of DNA linkers with 5′-OH ends, oligo 11 of linker F were phosphorylated by using [γ-32P] ATP and T4 DNA ligase. The kinase assay for E. coli DNA ligase was not performed because NAD^+^ labeled with 32P was not available for us. The phosphorylation of oligo 11 by T4 DNA ligase was performed in 25 µl of phosphorylation mixture containing 40 mM Tris-HCl (pH 8.0), 10 mM MgCl_2_, 10 mM DTT, 0.6 µCi/µl of [γ-32P] ATP, 4 µM of oligo 11, and 0.5 Weiss units/µl of T4 DNA ligase (Fermentas). 2 negative controls and 1 positive control were set for each reaction. One of 2 negative controls was without oligo 11 and another one was without ligase. The positive control was oligo 11 phosphorylated by T4 PNK. 25 µl of the positive control mixture contained 1 x PNK buffer A (50 mM Tris-HCl, pH 7.6 at 25°C, 10 mM MgCl_2_, 5 mM DTT, 0.1 mM spermidine and 0.1 mM EDTA), 2 µM of oligo 11, 0.6 µCi/µl of [γ-32P] ATP, and 0.6 U/µl of T4 PNK (Fermentas). These mixtures were incubated at 37°C for 1–2 hrs. The positive control was then diluted 10-fold with 1 x TE. Added 25 µl of 1 x TE and 5 µl of 10% SDS to the phosphorylation products generated by T4 DNA ligase and the negative controls, and then, extracted twice with an equal volume of phenol/chloroform (1∶1), and once with chloroform/isopentyl alcohol (24∶1). DNA was precipitated with 3 volumes of 100% ethanol and 1/10th volume of sodium acetate at −20°C for 10 hrs. DNA pellets were washed 3 times with 300 µl of 75% ethanol, dried at room temperature for about 2 hrs and resuspended in 4 µl of ddH_2_O. 4 µl of the resuspended DNA were mixed with 0.5 µl of 6 x DNA loading dye (Fermentas), and 0.5 µl of the positive control diluted 10-fold were mixed with 2 µl of ddH_2_O and 0.5 µl of 6 x DNA loading dye (Fermentas). These mixtures were then loaded on a 15% denaturing PAGE gel (10×10×0.03 cm, A:B  = 29∶1, 7 M urea, 0.5 x TBE). Electrophoresis was run in 0.5 x TBE at 100 V and 25°C for 3 hrs. The gel was fixed with 50 ml of 50% methanol containing 10% acetic acid for about 2 hrs, washed twice with 250 ml of dH_2_O for 10 min, and then dried between two semipermeable cellulose acetate membranes at 45°C for 2–3 hrs, and radioautographed at −20°C for 1–3 days.

To check if the phosphorylation of oligo 11 by T4 DNA ligase could be inhibited by CIAP treatment, oligo 11 was incubated with CIAP in 100 µl of CIAP reaction mixture containing 1 x CIAP buffer (50 mM of Tris-HCl, pH 9.0, 1 mM of MgCl_2_), 2 µM of oligo 11, and 0.4 U/µl of CIAP (Takara), at 37°C for 30 min, added 2 µl of 0.25 M EDTA, pH 8.0, and then inactivated at 85°C for 15–60 min. The CIAP reaction mixtures were extracted twice with phenol/chloroform (1∶1), and once with chloroform/isopentyl alcohol (24∶1). DNA was precipitated with 3 volumes of 100% ethanol and 1/10th volume of sodium acetate at −20°C for 10 hrs. DNA pellets were washed 3 times with 300 µl of 75% ethanol, dried at room temperature for about 2 hrs and resuspended in 4 µl of ddH_2_O. Then, the resuspended oligo 11 were phosphorylated by using T4 DNA ligase and radioautographed as described above.

## Results

### Ligation Products of the Complementary Linkers

**Figure 2:**
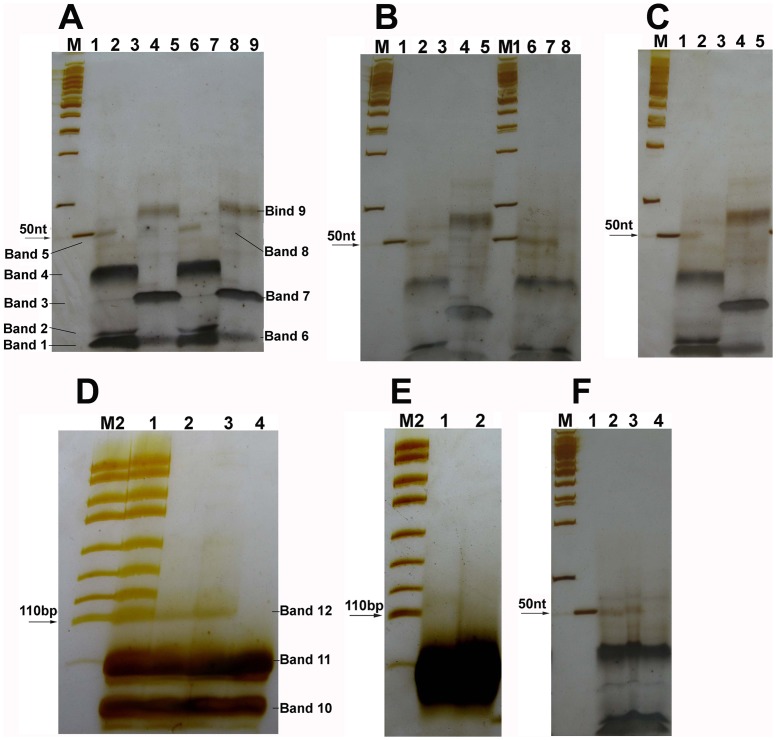
12% denaturing PAGE for the ligation products of linkers A–B, C–D, and E–F. PAGE (10×10×0.03 cm, A:B = 19∶1, 7 M urea and 0.5 x TBE) was run in 0.5 x TBE, 25°C, 200 V for 1.7 hrs for the ligation products of linkers A–B and C–D, or 100 V for 3.5 hrs for those of linkers E–F. The arrows indicate the ligation products. Lane M: DNA marker I (GeneRuler™ 50 bp DNA ladder, Fermentas); Lane M1: DNA marker I +1 µl of 1 µM oligo 15; Lane M2: pUC19 DNA/MspI Marker (Fermentas). (**A**) The ligation products joined by using T4 DNA ligase from Takara and Fermentas. Lane 1∶1 µl of 1 µM oligo 15; Lanes 2 and 6: the ligation products of linkers A–B joined by using T4 DNA ligase from Takara and Fermentas, respectively. We could see 5 bands. Of them, bands 1 and 2 were from oligos 4 and 1, respectively. Band 3 was from both oligos 2 and 3. Band 4 was unknown. Perhaps it might be the intermixtures of oligos 1–4. Band 5 was the denatured ligation products of linkers A–B; Lanes 4 and 8: the ligation products of linkers C–D joined by using T4 DNA ligase from Takara and Fermentas, respectively. We could see 4 bands. Of them, bands 6 and 7 were from both oligos 6 and 7, and both oligos 5 and 8, respectively. Band 8 was the denatured ligation products of linkers C–D. Band 9 was unknown. Perhaps it might be the intermixtures of oligos 5–8 and the double-strand ligation products of linkers C–D; Lanes 3, 5, 7, and 9: the negative controls. (**B**) The ligation products of linkers A–B and C–D joined by using T4 DNA ligase from Promega and the ligation products of linkers A–B joined in the ligase reaction mixture containing (NH_4_)_2_SO_4_. Lane 1∶1 µl of 1 µM oligo 15; Lanes 2 and 4: the denatured ligation products of linkers A–B, and C–D, respectively. T4 DNA ligase was from Promega; Lanes 6 and 7: the ligation products of linkers A–B joined in the ligase reaction mixture without (NH_4_)_2_SO_4_ and with (NH_4_)_2_SO_4_, respectively. T4 DNA ligase used was from Takara; Lanes 3, 5, and 8: the negative controls. (**C**) The ligation products of linkers A–B and C–D joined by using E. coli DNA ligase. Lane 1∶1 µl of 1 µM oligo 15; Lanes 2 and 4: the ligation products of linkers A–B, and C–D, respectively; Lanes 3 and 5: the negative controls. (**D**) The ligation products of linkers E–F joined in the ligase reaction mixture with (NH_4_)_2_SO_4_. The ligase was T4 DNA ligase (Fermentas). Lane 1: pUC19 DNA/MspI Marker plus 2 µl of ligation products of linkers E–F; Lanes 2 and 3: the ligation products of linkers E–F joined in the ligase reaction mixtures with (NH_4_)_2_SO_4_, and without (NH_4_)_2_SO_4_, respectively. We could see 3 bands. Bands 10 and 11 are from both oligos 9 and 12, and both oligos 10 and 11, respectively; Band 12 is the ligation products of linkers E–F; Lane 4: the negative control. (**E**) The ligation products of linkers E–F joined by using E. coli DNA ligase. Lane 1: the ligation products of linkers E–F. Lane 2: the negative control. (**F**) The ligation products of linkers A–B preincubated with T4 PNK in the E. coli DNA ligase reaction mixture without ATP. The ligase was E. coli DNA ligase (Takara). Lane 1∶1 µl of 1 µM oligo 15; Lane 2: linkers A–B were not preincubated with T4 PNK; Lane 3: linkers A–B were preincubated with T4 PNK; Lane 4: the negative control.

**Figure 3:**
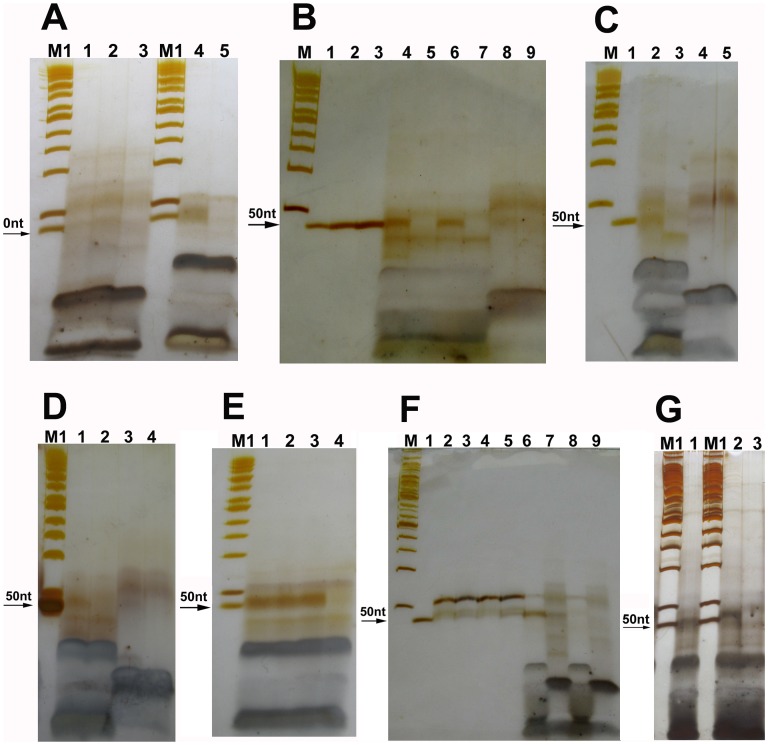
15% denaturing PAGE for the ligation products of linkers A–B, C–D and linkers G–H. PAGE (10×10×0.03 cm, A:B = 29∶1, 7 M urea, 0.5x TBE) was run in 0.5 x TBE, 25°C, 100 V for 3.5 hrs in (**A**)–(**F**), or 4.3 hrs in (**G**). The ligation products were indicated by the arrows. Lane M: DNA marker I (GeneRuler™ 50 bp DNA ladder, Fermentas). Lane M1: DNA marker I plus oligo 15. (**A**) The ligation products joined by using T4 DNA ligase from Fermentas. Lane 1: the ligation products of linkers C–D preincubated with T4 DNA ligase; Lane 2: the ligation products of linkers C–D without the preincubation; Lane 4: the ligation products of linkers A–B; Lanes 3 and 5: the negative controls. (**B**) The ligation products joined by using T4 DNA ligase from Takara. Lanes 1–3∶0.5, 1, and 2 µl of 1 µM oligo 15, respectively; Lanes 4 and 6: the ligation products of linkers A–B; Lane 8: the ligation products of linkers C–D. Lanes 5, 7, and 9: the negative controls. (**C**) The ligation products joined by using T4 DNA ligase from Promega. Lane 1∶1 µl of 1 µM oligo 15; Lanes 2 and 4: ligation products of linkers A–B, and C–D, respectively; Lanes 3 and 5: the negative controls. (**D**) The ligation products joined by using E. coli DNA ligase from Takara. Lanes 1 and 3: the ligation products of linkers A–B, and C–D, respectively; Lanes 2 and 4: the negative controls. (**E**) The ligation products of linkers A–B joined in T4 DNA ligase reaction mixture containing (NH_4_)_2_SO_4_. Lanes 1–3: the ligase reaction mixture with 7.5 mM (NH_4_)_2_SO_4_, 3.75 mM (NH_4_)_2_SO_4_, and without (NH_4_)_2_SO_4_, respectively; Lane 4: the negative control. (**F**) The ligation products of the phosphorylated linkers A–B and C–D joined by using T4 and E. coli DNA ligase (Takara). Lane 1∶1 µl of 1 µM oligo 15; Lanes 2 and 4: the ligation products of the phosphorylated linkers A–B joined by using T4 and E. coli DNA ligase, respectively; Lanes 3 and 5: the ligation products of the phosphorylated linkers C–D joined by using T4 and E. coli DNA ligase, respectively; Lanes 6 and 7: the ligation products of linkers A–B and C–D, respectively; Lanes 8 and 9: the negative controls of lanes 6 and 7, respectively. (**G**) The ligation products of linkers A–B and the phosphorylated linkers G–H. Lanes 1 and 2: the ligation products of linkers A–B and the ligation products of the phosphorylated linkers G–H plus the negative control of linkers A–B, respectively; Lane 3: the negative control of linkers G–H plus the negative control of linkers A–B. The band from the ligation products of the phosphorylated linkers G–H run a little more slowly than that of linkers A–B. The sequences of linkers G and H are similar to those of linkers A and B, respectively. But there is a 1-base deletion at the 5′ end of each of linkers G and H.

**Figure 4:**
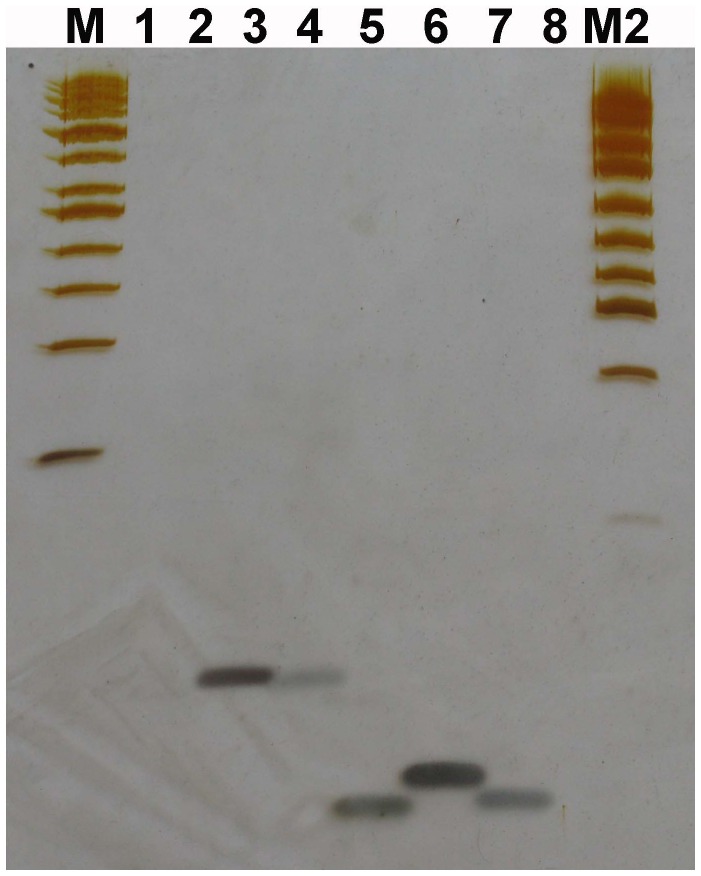
15% denaturing PAGE for oligos 1–8. PAGE (10×10×0.03 cm, A:B = 29∶1, 7 M urea, 0.5 x TBE) was run in 0.5 x TBE buffer at 25°C and 100 V for 3.5 hrs. The silver stain method was described in the text. Lanes M and M2: DNA marker I (GeneRuler™ 50 bp DNA ladder, Fermentas) and pUC19 DNA/MspI Marker (Fermentas), respectively. Lanes 1–8: oligos 2, 3, 5, 8, 4, 1, 6, and 7, respectively. Of them, oligos 2, 3, and 7 could not be fixed with 50% methanol containing 10% acetic acid and silver-stained.

To explore if the linkers with 5′-OH ends could be joined by commercial T4 and E. coli DNA ligase, linkers A–B, C–D, and E–F were joined by using commercial T4 or E. coli DNA ligase. Their ligation products could be detected by using 12% and 15% denaturing PAGE (Figures 2 and 3). In Figure 2, there are 5 bands (bands 1–5), 4 bands (bands 6–9), and 3 bands (bands 10–12) in the lanes of the ligation products of linkers A–B, C–D, and E–F, respectively. To characterize these bands, we run a denaturing PAGE for all the oligos of linkers A–B and C–D. The result was showed in Figure 4. Basing on this result and the lengths of these oligos, we inferred that bands 1 and 2 were from oligos 4 and 1, respectively. Band 3 was from both oligos 2 and 3. Band 4 was unknown. Perhaps it might be the intermixtures of oligos 1–4. Band 5 was the denatured ligation products of linkers A–B. Bands 6 and 7 were from both oligos 6 and 7, and both oligos 5 and 8, respectively. Band 8 was the denatured ligation products of linkers C–D. Band 9 was unknown. Perhaps it might be the intermixtures of oligos 5–8 and the double-strand ligation products of linkers C–D. Bands 10 and 11 were from both oligos 9 and 12, and both oligos 10 and 11, respectively. Band 12 was the ligation products of linkers E–F. Band 3 from oligos 2 and 3 of linkers A–B was very weak. We supposed that the bands 5, 8, and 12 were from the ligation products of linkers A–B, C–D, and E–F, respectively, because: (i) their lengths were consistent with the expectation; and (ii) there were no these bands in the negative controls. Based on the band density of the ligation products and oligo 15 used as a marker, we estimated roughly that about 0.5–1% of linkers A–B and E–F, and 0.13–0.5% of linkers C–D could be joined by T4 DNA ligases. About 0.25–0.77% of linkers A–B and E–F, and 0.06–0.39% of linkers C–D could be joined by E. coli DNA ligases. To figure out if the ligation of linkers A–B and E–F could be inhibited by (NH_4_)_2_SO_4_, a strong inhibitor of T4 PNK, the ligation of these linkers were performed in T4 DNA ligase reaction mixture containing 7.5 mM (NH_4_)_2_SO_4_. The results showed that the ligation of linkers A–B and E–F could not be significantly inhibited by (NH_4_)_2_SO_4_ (Figures 2B and D, and Figure 3E). To see if T4 PNK could use NAD^+^ as its phosphate donor, oligos 2 and 3 of linkers A–B were separately preincubated with T4 PNK in the reaction mixture with NAD^+^, but without ATP. Then these linkers were joined by using E. coli DNA ligase. The ligation results demonstrated that the ligation yield of linkers A–B preincubated with T4 PNK in the reaction mixture containing NAD^+^ did not increased, indicating that T4 PNK could not use NAD^+^ as its phosphate donor (Figure 2F).

**Figure 5:**
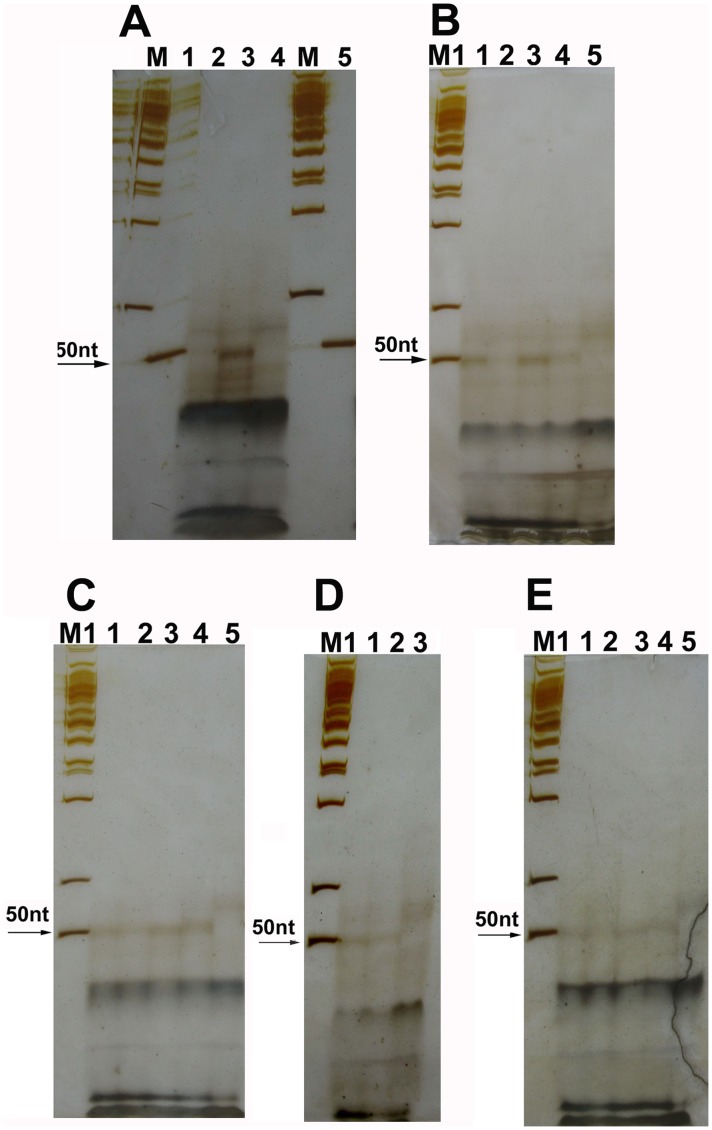
12% denaturing PAGE for the ligation products of linkers A–B treated with CIAP. PAGE (10×10×0.03 cm, A:B  = 19∶1, 7 M urea and 0.5 x TBE) was run in 0.5 x TBE, 25°C, 200 V for 1.7 hrs. The arrows indicate the ligation products. Lane M: DNA marker I (GeneRuler™ 50 bp DNA ladder, Fermentas); Lane M1: DNA marker I +1 µl of 1 µM oligo 15. The ligases used in (**A**)–(**C**) were T4 DNA ligases. The ligases used in (**D**)–(**E**) were E. coli DNA ligases. (**A**) CIAP was inactivated at 75°C for 15 min. Lanes 1 and 5∶1 µl of 1 µM oligo 15; Lanes 2: CIAP was inactivated at 75°C for 15 min; Lane 3: the positive control without CIAP treatment; Lane 4: the negative control without ligase. (**B**) CIAP was inactivated at 85°C for 25 min and 45 min. Lanes 1 and 3: the positive controls without CIAP treatment; Lanes 2 and 4: CIAP was inactivated at 85°C for 25 min and 45 min, respectively; Lane 5: the negative control without ligase. (**C**) CIAP was inactivated at 85°C for 65 min and 90 min. Lanes 1 and 3: the positive controls without CIAP treatment; Lanes 2 and 4: CIAP was inactivated at 85°C for 65 min and 90 min, respectively; Lane 5: the negative control without ligase. (**D**) CIAP was inactivated at 85°C for 45 min. Lanes 1 and 3: the positive control without CIAP treatment and the negative control without ligase, respectively; Lane 2: CIAP was inactivated at 85°C for 45 min. (**E**) CIAP was inactivated at 85°C for 65 and 90 min. Lanes 1 and 3: the positive controls without CIAP treatment; Lanes 2 and 4: CIAP was inactivated at 85°C for 65 and 90 min, respectively; Lane 5: the negative control without ligase.

To compare the ligation products between the linkers with 5′-phosphate ends and the linkers with 5′-OH ends, linkers A–B, C–D and G–H were separately phosphorylated by using T4 PNK, and then joined by using T4 and E. coli DNA ligase. As a result, we could see that the ligation products of linkers with 5′-OH ends were similar, but not completely, to those of the phosphorylated linkers (Figure 3F). Based on the band density, we could estimated that more than half of the ligation products of linkers A–B with 5′-OH ends were single strands, while more than half of those of the phosphorylated linkers A–B were double strands. The ligation products of linkers A–B were also similar to those of the phosphorylated linkers G–H which deleted a nucleotide at their 5′-ends (Figure 3G). Therefore, we speculated that some of the ligation products of linkers A–B might also be generated from the linkers with one or more nucleotide deletion(s) at their 5′-ends. To see if the ligation of linkers A–B could be inhibited by CIAP treatment, oligos 2 and 3 of linkers A and B were treated with CIAP before ligation. As a result, the ligation products of linkers A–B treated with CIAP reduced obviously when CIAP was inactivated at 85°C for only 15–25 min (Figures 5A–B). The ligation products increased again when CIAP was inactivated at 85°C for more than 30 min (Figures 5B–E). These results of CIAP treatments might perhaps give us at least 3 messages: (i) CIAP treatments of linkers A–B could block, but not completely, the ligation of linkers A–B, indicating that some of linkers A–B could still be joined by T4 DNA ligase even after CIAP treatment; (ii) the ligation of linkers A–B could be recovered after CIAP was inactivated at 85°C for 30–90 min, perhaps suggesting that some of linkers A–B could spontaneously delete one or more nucleotide(s) at their 5′-ends and generate some 5′-phosphate ends when the linkers were incubated at 85°C for 30–90 min; and (iii) these results of CIAP treatments again indicated that band 5 in Figure 2A was the ligation products of linkers A–B.

### Three Round Overlap PCR Products and DNA Sequencing

**Figure 6:**
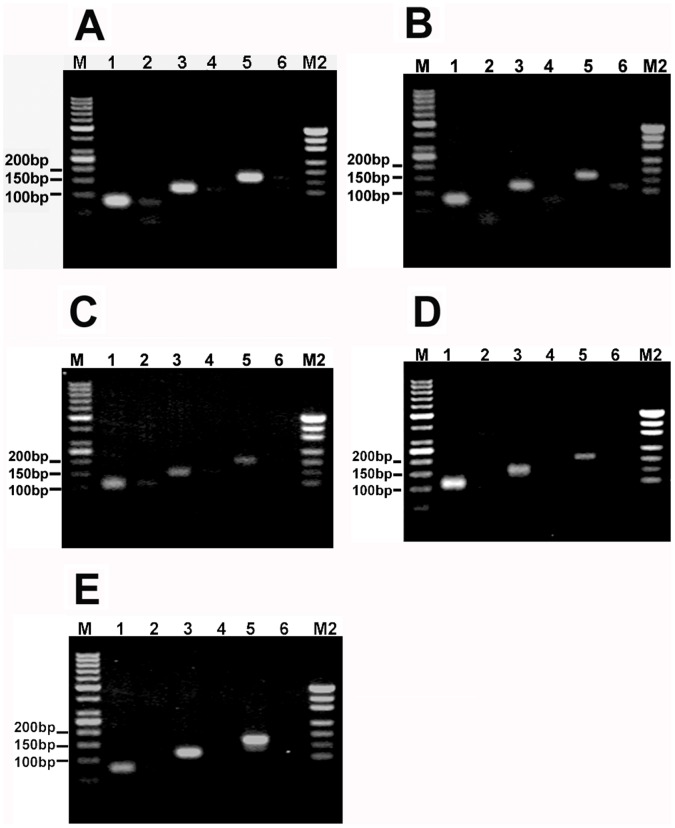
2.5% agarose gel electrophoreses for the three round PCR products. Electrophoreses were run in 1 x TAE at 60 V for 40 min. Lanes M and M2: DNA marker I and pUC19 DNA/MspI Marker, respectively; Lanes 1, 3, and 5: the first, second, and third round PCR products, respectively; Lanes 2, 4, and 6: the negative controls. (**A**) and (**B**)The first round PCR templates were the ligation products of linkers A–B joined by T4 and E. coli DNA ligases, respectively. (**C**) and (**D**) The first round PCR templates were the ligation products of linkers C–D joined by T4 and E. coli DNA ligases, respectively. (**E**) The first round PCR templates were the ligation products cut from the denaturing PAGE gel.

**Figure 7:**
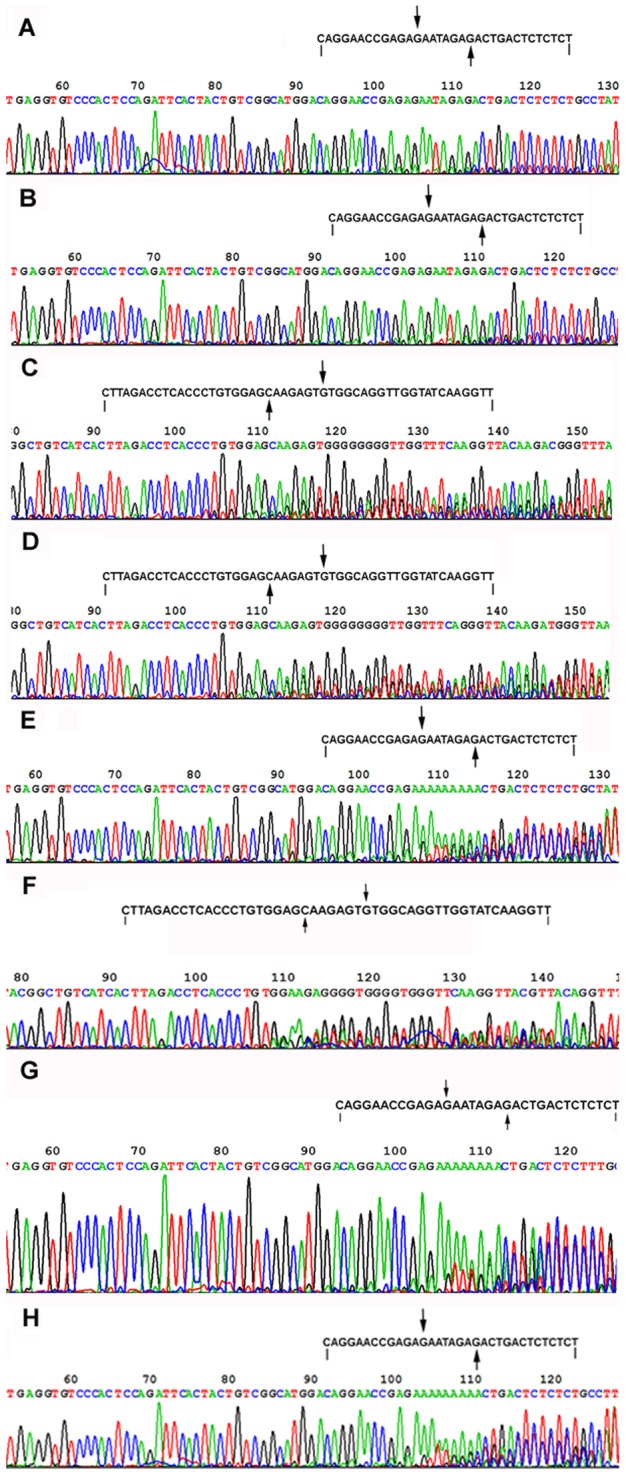
DNA sequencing results of the third round PCR products. The letters on the top are the expected DNA sequences. The downward arrows and the upward arrows indicate the ligation junctions of the sense strands and the antisense strands, respectively. (**A**) and (**B**) The sequencing templates were prepared from the ligation products of linkers A–B joined by T4 and E. coli DNA ligases, respectively. The ligation products were purified by using a PCR product purification kit before PCR. There was a 1-base deletion (-G) at the ligation junctions of both sense and antisense strands. The signal intensity from these deletions was only equivalent to about 25% of that from the normal sequences. (**C**) and (**D**) The sequencing templates were prepared from the ligation products of linkers C–D by T4 and E. coli DNA ligases, respectively. The ligation products were purified by using a PCR product purification kit before PCR. A 5-base deletion (-GGAGC) was found at the ligation junction of the antisense strand. The signal intensity from the deletion was only equivalent to about 25% of that from the normal sequence. (**E**) and (**F**) DNA sequencing template was prepared from the unpurified ligation products of linkers A–B and C–D, respectively. A 1-base deletion (-G) or a 5-base deletion (-GGAGC) was found at the ligation junctions of both sense and antisense strands of linkers A–B, or the ligation junction of the antisense strand of linkers C–D, respectively. The signal intensity from these deletions was equivalent to or even stronger than that from the normal sequence. (**G**) DNA sequencing template was prepared from the ligation products of linkers A–B cut from the denaturing PAGE gel. There was a 1-base deletion (-G) at the ligation junctions of both sense and antisense strands. (**H**) DNA sequencing template was prepared from the negative control of linkers A–B cut from the denaturing PAGE gel. There was 1-base deletion (-G) at the ligation junctions of both sense and antisense strands.

To prepare the sequencing template, the ligation products of linkers A–B and C–D were amplified by using 3 round overlap PCR. The 3 round overlap PCR products from the ligation products of linkers A–B or linkers C–D were 78, 121, and 165 bp long, or 109, 148, and 201 bp long, respectively. These PCR products were checked by using 2.5% agarose gel. All of them could be detected by using 2.5% agarose gel electrophoresis (Figure 6), again indicating that the linkers with 5′-OH ends could be joined by T4 and E. coli DNA ligases. To see if the linkers with 5′-OH ends could be correctly joined by T4 and E. coli DNA ligase, the third round PCR products were analyzed by DNA sequencing. The sequencing results revealed that linkers A–B and C–D had been joined by T4 or E. coli DNA ligase (Figure 7). We could find a 1-base deletion (-G) at the ligation junctions of both sense and antisense strands of linkers A–B, and a 5-base deletion (-GGAGC) at the ligation junction of the antisense strand of linkers C–D. The signal intensity from these deletions was only equivalent to about 25% of that from the normal sequences if the ligation products of linkers A–B and C–D were purified with a PCR product purification kit before PCR (Figures 7A–D), otherwise, the signal intensity from these deletions was equivalent to or even stronger than that from the normal sequences if the ligation products of linkers A–B and C–D were not purified with a PCR product purification kit before PCR (Figures 7E–G). These sequencing results indicated that: (i) about 80% (calculated according to the formula: the signal intensity from the ligation products without base deletion + the signal intensity from the ligation products with base deletion  = 1, namely, 1÷1.25×% = 80%) of the ligation products purified with a PCR product purification kit did not contain these base deletions, meaning that some linkers had been correctly joined by T4 and E. coli DNA ligases; (ii) the ligation products of the linkers phosphorylated by T4 DNA ligase could be selectively collected by the PCR product purification kit; and (iii) perhaps these base deletions might be partly caused by PCR as well as by the base deletions at the 5′-ends of the linkers. Therefore, it was difficult for us to estimate how many ligation products did not contain these base deletions if these ligation products were not purified with a PCR product purification kit. If only based on the signal intensity from Figures 7E–F, we could estimate that about 30–50% of the unpurified ligation products did not contained base deletions. The sequencing result of the negative control of linkers A–B showed that there was the same base deletion (-G) at the ligation junction (Figures 7H), again indicating that this base deletion might be partly caused by PCR.

### Kinase Assay

**Figure 8:**
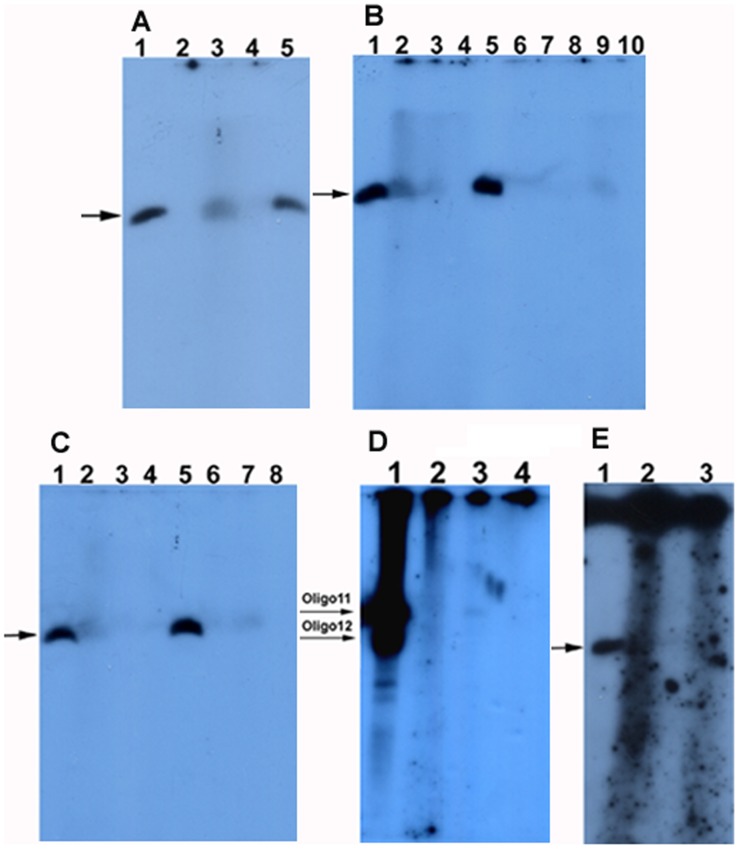
The radioautograph of oligo 11 phosphorylated by T4 DNA ligase. The oligo 11 was phosphorylated by using commercial T4 DNA ligase. The phosphorylation products were loaded on a 15% denaturing PAGE gel (10×10×0.03 cm, A:B  = 29∶1, 7 M urea, 0.5 x TBE). Electrophoresis was run in 0.5 x TBE at 100 V and 25°C for 3 hrs. The gel was dried between two semipermeable cellulose acetate membranes and radioautographed at −20°C for 1–3 days. The arrows indicate the phosphorylation products. The positive controls were oligo 11 phosphorylated by T4 PNK. (**A**) Oligo 11 was phosphorylated by T4 DNA ligase at 37°C for 2 hrs. Lanes 1 and 5: the positive controls; Lanes 2 and 4: the negative controls without ligase, and without oligo 11, respectively; Lane 3: the phosphorylation products of oligo 11 by T4 DNA ligase. (**B**) Oligo 11 treated with CIAP was phosphorylated by T4 DNA ligase at 37°C for 2 hrs. Lanes 1 and 5: the positive controls; Lane 2: the phosphorylation products of oligo 11 by T4 DNA ligase; Lanes 3 and 4: the negative controls without ligase, and without oligo 11, respectively; Lanes 6, 7, and 8: oligo 11 treated with CIAP was phosphorylated by T4 DNA ligase. CIAP was inactivated at 85°C for 15 min, 30 min, and 60 min, respectively. Lanes 9 and 10: the negative controls without ligase, and without oligo 11, respectively. (**C**) Oligo 11 treated with CIAP was phosphorylated by T4 DNA ligase at 37°C for 2 hrs. Lanes 1 and 5: the positive controls; Lane 2: the phosphorylation products of oligo 11 by T4 DNA ligase; Lanes 3 and 4: the negative controls without ligase, and without oligo 11, respectively; Lanes 6, 7, and 8: oligo 11 treated with CIAP was phosphorylated by T4 DNA ligase. CIAP was inactivated at 85°C for 60 min, 15 min, and 30 min, respectively. (**D**) Oligos 11 and 12 were phosphorylated by T4 DNA ligase at 37°C for 1 hr. Lane 1: oligos 11 and 12 were phosphorylated by T4 PNK; Lane 2: oligos 11 and 12 were phosphorylated by T4 DNA ligase; Lane 3: oligo 11 were phosphorylated by T4 DNA ligase; Lane 4: the negative control without ligase. (**E**) Oligo 11 was phosphorylated by T4 DNA ligase at 37°C for 2 hrs. 1 x TE and 10% SDS were not added to the phosphorylation products before phenol/chloroform extraction. Lane 1: the positive control; Lanes 2 and 3: the phosphorylation products of oligo 11 by T4 DNA ligase and the negative controls without ligase, respectively.

To explore the ligation mechanism of DNA linkers with 5′-OH ends by T4 DNA ligase, oligo 11 of linker F were phosphorylated by using [γ-32P] ATP and T4 DNA ligase. As a result, the phosphorylation products of oligo 11 could be detected by radioautography, suggesting that the 5′-OH of oligo 11 could be phosphorylated by T4 DNA ligase (Figure 8). Based on the band density of the phosphorylation products, we estimated roughly that about 0.025–0.1% of oligo 11 could be phosphorylated by T4 DNA ligase. The phosphorylation products would increase when the phosphorylation reaction at 37°C was extended from 1 hr to 2 hrs (comparing Figures 8A–C with Figure 8D). Combining this result with that of DNA sequencing, we supposed that the ligation products of the linkers with 5′-OH ends might be the intermixtures of the ligation products of the linkers phosphorylated by T4 DNA ligase and those of the linkers which deleted some nucleotides at their 5′-ends. To check if the phosphorylation of oligo 11 by T4 DNA ligase could be inhibited by CIAP treatment, oligo 11 was treated with CIAP before it was phosphorylated by T4 DNA ligase. As a result, the phosphorylation products of oligo 11 by T4 DNA ligase reduced if oligo 11 was treated with CIAP (Figures 8B–C). The phosphorylation products by T4 DNA ligase were more when CIAP was inactivated at 85°C for 15 min than at 85°C for 30–60 min (Figures 8B–C). It was unknown why the phosphorylation of oligo 11 by T4 DNA ligase could be inhibited by CIAP treatment.

## Discussion

Our experiments showed that DNA linkers with 5′-OH ends could be joined by both T4 and E. coli DNA ligases. The ligation products could be detected directly by using 12% and 15% denaturing PAGE silver stain (Figures 2 and 3), or indirectly by using the first round PCR. We had to perform 3 round overlap PCR to generate DNA sequencing template because the first round PCR products were too short to be analyzed by direct DNA sequencing. DNA sequencing results showed a 1-base or 5-base deletion at the ligation junction between linkers A–B or C–D, respectively. We inferred that these deletions might be generated in 2 manners: (1) they were generated partly by the PCR because (i) the deletion background could be significantly reduced by the purification of the ligation products with a PCR product purification kit (Figures 7A–D); and (ii) when the second round PCR for the negative control of linkers A–B was run 15 cycles as many as those of the positive sample, the third round PCR products of the negative control were hardly detectable. However, when the second round PCR was run up to 25 cycles, the third round PCR products of the negative control were rich enough to be analyzed by DNA sequencing. The sequencing result for this negative control showed the same deletion as that in the ligation products of linkers A–B (Figure 7H); and (2) they might be generated partly by nucleotide deletion at the 5′-ends of the linkers. Since it was impossible that oligos with 5′-phosphate groups could be synthesized by using the solid-phase phosphoramidite method without any chemical modification, we inferred that the 5′-ends of linkers could delete one or more nucleotide(s) spontaneously or by the possibly contaminated nucleases.

It is quite clear that the ligation mechanism of DNA nicks or breaks with 5′-phosphate includes the following steps. First, DNA ligase is activated through the formation of a covalent protein-AMP intermediate. Second, the AMP moiety is transferred from the ligase to the 5′-phosphate group at the nick site. Finally, DNA ligase joins the DNA nick or break by catalyzing the formation of a phosphodiester bond between the adjacent 5′-phosphate and the 3′-OH ends with the release of AMP. However, it is unclear why these DNA linkers with 5′-OH ends could be joined by commercial T4 or E. coli DNA ligases. Based on our experimental results, we have 3 hypotheses. First, the 5′-OH ends of the DNA linkers might be first phosphorylated by T4 DNA ligase, and then these phosphorylated DNA linkers were joined by T4 DNA ligase through the three steps mentioned above. This hypothesis seems to be supported by our kinase assay showing that T4 DNA ligases had a weak kinase activity. Perhaps we could imagine that a ligase might facilitate the phosphorylation of oligos. In the ligase-ATP-Mg^2+^-DNA complex, the ligase may somewhat act as a kinase through the general mechanism of enzyme catalysis: the proximity and orientation effect. If so, the kinase activity perhaps is a minor one simply due to the fact that a ligase can place ATP or NAD^+^ to the proximity of the 5′-OH. Second, the DNA ligases used might contain trace amount of PNK. But this hypothesis seems not to be supported by (i) T4 and E. coli DNA ligase are polypeptides with molecular weights of 68 and 75 kDa, respectively. T4 PNK consists of four identical subunits of 28.9 kDa each. These enzymes should be able to be easily separated because their structures and molecular weights are quite different from each other; (ii) DNA ligases used were purchased from the professional manufacturers. When these manufacturers were questioned, they stated that their T4 DNA ligases had very high quality and it was very unlikely that there would be PNK in their ligases because their ligases were produced by using E. coli cells and production lines that were different from those for T4 PNK. A quality inspection report of T4 DNA ligase from Fermentas showed that T4 PNK could not be detected in their T4 DNA ligase (Supporting information S1); (iii) PNK could not be detected in T4 DNA ligase (Fermentas) by using mass spectrometry (MS) analysis (Supporting information S2 and S3); (iv) PNK is abundant in mammalian cells but absent in E. coli cells [14]. Therefore, the endogenous PNK should be absent in the host E. coli cells that carry plasmids enabling T4 or E. coli DNA ligase high expression; (v) The ligation of linkers A–B and E–F could not be significantly inhibited by (NH_4_)_2_SO_4_, a strong inhibitor of T4 PNK (Figures 2B and D, and Figure 3E); and (vi) T4 PNK requires ATP for activity. Our experiment showed that T4 PNK could not use NAD^+^ as its phosphate donor. However, both Type I and II linkers could be joined by E. coli DNA ligases even if there was no ATP in the E. coli DNA ligase reaction mixture (Figures 2C and F, and Figure 3D). Third, the 5′-ends of the linkers could delete some nucleotides spontaneously or by the possibly contaminated nucleases, and thereby formed some 5′- phosphate ends. Although the efficiency was low, now the ligase could join the newly appeared 5′-phosphate to the 3′-OH of the other linkers, which resulted in the amplification of ligation product with a nucleotide deletion at the junction point.

The ligation products of linkers A–B could be reduced by CIAP treatment of the linkers when CIAP was inactivated at 85°C for 15–25 min, but they could increase again when CIAP was inactivated at 85°C for 30–90 min. The reasons were unknown. We presumed that the linkers with 5′-phosphate generated by a spontaneous nucleotide deletion would increase when the incubation at 85°C was extended. Therefore, the ligation products would decrease when the 5′-phosphate generated by the spontaneous nucleotide deletion was removed by CIAP, but increase again when the linkers deleting one or more nucleotide(s) at their 5′-ends increased as the CIAP inactivation at 85°C was extended from 15 min to 30–90 min.

Our kinase assay for T4 DNA ligase showed that about 0.025–0.1% of oligo 11 could be phosphorylated by T4 DNA ligase and the phosphorylation of oligo 11 by T4 DNA ligase could be inhibited by CIAP treatment of oligo 11. The phosphorylation products by T4 DNA ligase were more when CIAP was inactivated at 85°C for 15 min than at 85°C for 30–60 min. It was unknown why the phosphorylation of oligo 11 could be inhibited by CIAP treatment. We supposed that the reasons might include: (i) CIAP mixture perhaps contained some inhibitors from CIAP or the other components of CIAP mixture; and (ii) an oligo might be hard to be phosphorylated by T4 DNA ligase if it were incubated in CIAP mixture at 85°C for more than 15 min. Our DNA sequencing results demonstrated one or more base deletion(s) at the ligation junction between linkers, but the base deletion background could be significantly reduced if the ligation products of linkers A–B and C–D were purified with a PCR product purification kit before PCR. These results might suggest that the ligation products of the linkers phosphorylated by T4 DNA ligase could be selectively collected by the PCR product purification kit. Since the base deletion background of the ligation products generated by E. coli DNA ligase could also be significantly reduced by the purification of the ligation products with a PCR product purification kit, we speculated that the ligation products generated by E. coli DNA ligase might also contain the ligation products of the phosphorylated linkers, or in other words, perhaps the linkers with 5′-OH ends could be phosphorylated by E. coli DNA ligase, too.

**Figure 9:**
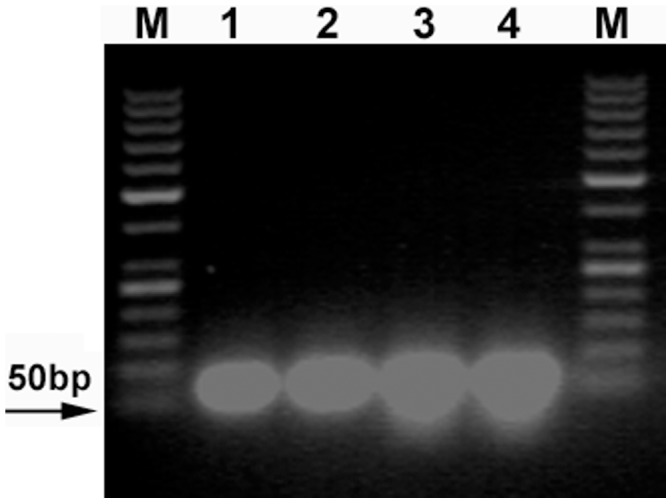
2.5% agarose gel electrophoresis for the ligation products of linkers A–B and C–D. The gel contained 0.5 µg/ml of EB. 20 µl of the original ligation products were loaded to each well. Electrophoresis was run in 1 x TAE, at 60 V for 40 min. Lane M: DNA marker I; Lanes 1 and 3: the ligation products of linkers A–B, and C–D, respectively. Lanes 2 and 4: the negative controls of lanes 1 and 3, respectively.

T4 and E. coli DNA ligases have been deeply researched and widely used as molecular biology tools since they were discovered a long time ago. It is unclear why the ligation products of DNA linkers with 5′-OH ends joined by T4 or E. coli DNA ligase are rarely reported. We speculated that the reasons might include: (i) in the past, the ligation products loaded on a gel were too little to be detected by silver stain because the maximum volume of a 0.3-mm-thin gel was about 10 µl if the wells were created by a shark tooth comb. Of course, the maximum volume of a well could be increased if a thicker gel was used. But the silver stain for a thick gel would be difficult to be done. It was because the ligation products were enriched 12.5 to 25 fold that the ligation products could be detected by us. Or in other words, the enriched ligation products loaded to each well by us was equivalent to 50–100 µl of the unenriched ones. Therefore they could be detected by us; (ii) the ligation products could not be separated from the ligation substrates (the oligos of linkers) by using an agarose gel containing EB (Figure 9). Moreover, the sensitivity of EB was lower than that of silver stain; and (iii) the sensitivity of radioautograph may perhaps be higher than that of silver stain, but the signals from ligation products could be covered by those from the substrates (the oligos of linkers) because the latter would be much stronger than the former (refer Figure 8D). In addition, it was the most important that we added 1 x TE and 10% SDS to the phosphorylation products generated by T4 DNA ligase before they were extracted with phenol/chloroform, otherwise, the signals from the phosphorylation products would be covered by those from the background (Figure 8E).

In the previous experiments [15], we found that the short DNA fragments (<80 nt) were hard to be fixed with 10% acetic acid. Therefore, in this experiment, we fixed the ligation products and the oligos of linkers with 50% methanol containing 10% acetic acid. The ligation products and most oligos of the linkers could be fixed by this fixing solution, but oligos 2, 3, and 7 could not be well fixed (Figures 2, 3, and 4) and silver stained. The reason is unknown. We found that the oligos (20–30 nt long) could be fixed with 50% methanol containing 10% acetic acid and silver-stained if their base compositions could meet either of the following 2 conditions: (i) the amount of base A ≥8; and (ii) the amount of base G ≥7 if the amount of base A ≤7, but ≥5 (Table 3).

**Table 3:** Base compositions of the oligos of the linkers and silver-stain.

Linker	Oligo	Length (nt)	Base compositions (nt; %)				Silver stain*
			A	C	G	T	
A	1	22	7;31.8	5;22.7	8;36.4	2;9.1	+
	2	30	3;10.0	11;36.7	5;16.7	11;36.7	−
B	3	28	7;25.0	7;25.0	5;17.9	9;32.1	−
	4	20	8;40.0	2;10.0	7;35.0	3;15.0	+
C	5	29	7;24.1	8;27.6	8;27.6	6;20.7	+
	6	21	5;23.8	5;23.8	7;33.3	4;19.0	+
D	7	21	4;19.0	2;9.5	8;38.1	7;33.3	−
	8	29	8;27.6	11;37.9	3;10.3	7;24.1	+

* (+)  = positive; (−)  = negative.

In conclusions, our experimental results indicate that about 0.5–1% of linkers A–B and E–F, and 0.13–0.5% of linkers C–D could be joined by commercial T4 DNA ligases. About 0.25–0.77% of linkers A–B and E–F, and 0.06–0.39% of linkers C–D could be joined by commercial E. coli DNA ligases. These ligation products could be detected directly by using denaturing PAGE silver stain, or indirectly by using PCR. There was a 1-base or 5-base deletion at the ligation junction between linkers A–B or C–D, respectively. But 80% of the ligation products purified with a PCR product purification kit did not contain these base deletions, meaning that some linkers with 5′-OH ends had been correctly joined by T4 and E. coli DNA ligases. CIAP treatments of linkers A–B could block, but not completely, the ligation of linkers A–B. About 0.025–0.1% of oligo 11 could be phosphorylated by commercial T4 DNA ligase. The phosphorylation products could be increased when the phosphorylation reaction was extended from 1 hr to 2 hrs. The phosphorylation of oligo 11 by T4 DNA ligase could be inhibited by CIAP treatment of oligo 11. The phosphorylation products by T4 DNA ligase were more when CIAP was inactivated at 85°C for 15 min than at 85°C for 30–60 min. We speculated that perhaps the linkers with 5′-OH could be joined by the commercial T4 or E. coli DNA ligase in 2 different manners: (i) the commercial T4 DNA ligase could phosphorylate the 5′-OH of linkers at a low efficiency, and then join them to the 3′-OH ends of other linkers; and (ii) the 5′-ends of the linkers could delete one or more nucleotide(s) spontaneously or by the possibly contaminated nucleases, and thereby generated some 5′-phosphate ends, and then these 5′-phosphate ends could be joined to the 3′-OH ends of other linkers at a low efficiency. Our findings may perhaps indicate that some DNA nicks with 5′-OH ends could be joined by T4 or E. coli DNA ligase even in the absence of PNK. But a base deletion mutation would be created if the nicks were joined in the manner of base deletion.

## Supporting Information

Supporting Information S1 **A quality inspection report of T4 DNA ligase from Fermentas.** This report showed that T4 PNK could not be detected in T4 DNA ligase.
(PDF)

Supporting Information S2 **A report of MS analysis for T4 DNA ligase (Fermentas).** This report showed that PNK could not be detected in T4 DNA ligase by using MS analysis.
(PDF)

Supporting Information S3 **A protocol for MS analysis of T4 DNA ligase.** This protocol describes the procedure of MS analysis of T4 DNA ligase (Fermentas).
(DOC)
