# Supplementary material for: Detection of Ligation Products of DNA Linkers with 5′-OH Ends by Denaturing PAGE Silver Stain
Source: PLoS One. 2012 Jun 27;7(6):e39251. doi: 10.1371/journal.pone.0039251 (PMC3384673; doi:10.1371/journal.pone.0039251)
Supplement: Supporting Information S1 — A quality inspection report of T4 DNA ligase from Fermentas. This report showed that T4 PNK could not be detected in T4 DNA ligase. (PDF) [file pone.0039251.s001.pdf]

Purity of EL0014 lot00032044

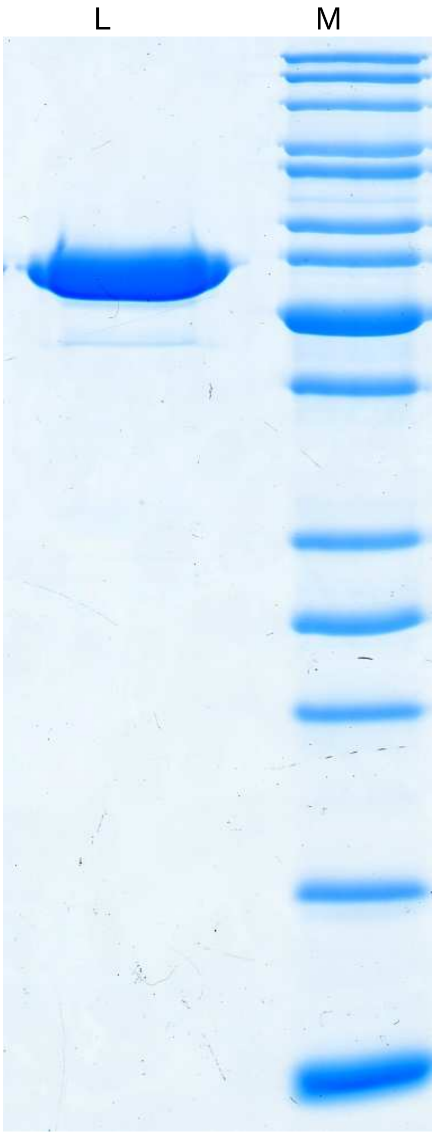

2 µg of T4 DNA ligase, concentration 0.538µg/ul (L) was run on 13% Tris-glycine SDS-PAGE.
M - 5 µl of PageRuler™ Unstained Protein Ladder (SM0661).

<u>Conclusion:</u> The purity of EL0014 lot00032044 is 99%.

Fermentas UAB
V.A. Graičiūno 8
LT-02241 Vilnius
Lithuania

Tel. +370 5 2602131
Fax +370 5 2602142
info@fermentas.lt
www.fermentas.com

Company data are submitted and stored at
Register of Legal Entities of Lithuanian Republic
Company Code 122351387
VAT Code LT223513811
