# Supplementary material for: Detection of Ligation Products of DNA Linkers with 5′-OH Ends by Denaturing PAGE Silver Stain
Source: PLoS One. 2012 Jun 27;7(6):e39251. doi: 10.1371/journal.pone.0039251 (PMC3384673; doi:10.1371/journal.pone.0039251)
Supplement: Supporting Information S2 — A report of MS analysis for T4 DNA ligase (Fermentas). This report showed that PNK could not be detected in T4 DNA ligase by using MS analysis. (PDF) [file pone.0039251.s002.pdf]

Mascot Search Results

```
User          :
Email         :
Search title  : lihongtao\20110901\MSMS 3\E1
Database      : UniProtKB-SwissProt sprot_20081216 (405506 sequences; 146166984 re
Timestamp     : 28 Oct 2011 at 08:44:54 GMT
Warning       : A Peptide summary report will usually give a much clearer picture
Top Score     : 809 for P00970, DNA ligase OS=Enterobacteria phage T4 GN=30 PE=3 S
```

Mascot Score Histogram

Protein score is -10*Log(P), where P is the probability that the observed match is a random event.
Protein scores greater than 69 are significant (p<0.05).
Protein scores are derived from ions scores as a non-probabilistic basis for ranking protein hits.

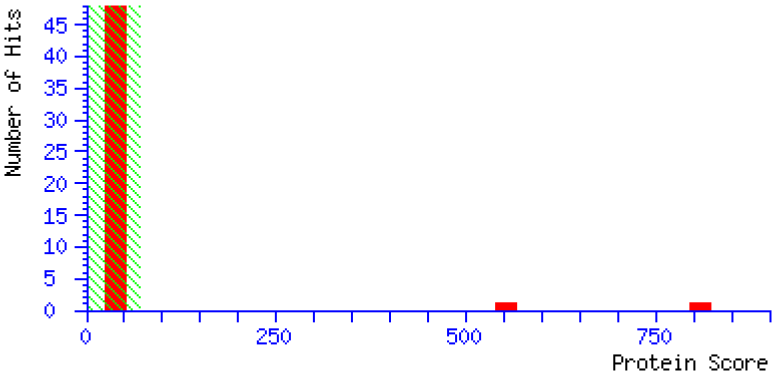

Protein Summary Report

Format As | Protein Summary (deprecated) | Help

Significance threshold p< 0.05 Max. number of hits AUTO

Re-Search All | Search Unmatched

Index

	Accession	Mass	Score	Description
1.	P00970	55600	809	DNA ligase OS=Enterobacteria phage T4 GN=30 PE=3 SV=1
2.	P19088	55574	556	DNA ligase OS=Enterobacteria phage T6 GN=30 PE=3 SV=1
3.	P28376	52059	61	Ribulose bisphosphate carboxylase large chain (Fragment)

Results List

1. P00970 **Mass:** 55600 **Score:** 809 **Expect:** 5.1e-076 **Matches:** 32

DNA ligase OS=Enterobacteria phage T4 GN=30 PE=3 SV=1

Observed	Mr(expt)	Mr(calc)	ppm	Start	End	Miss	Ions	Peptide
845.4611	844.4538	844.4290	29.3	100 -	106	0	---	K.DDVEVLR.R

884.5102	883.5029	883.4803	25.6	42 - 48	0	---	R.GLQYYIK.K
918.4479	917.4406	917.4090	34.4	171 - 178	0	---	R.GDELDDVR.L
921.5546	920.5473	920.5120	38.4	152 - 159	0	---	K.FPAFAQLK.A
921.5546	920.5474	920.5120	38.4	152 - 159	0	47	K.FPAFAQLK.A
942.6553	941.6480	941.6062	44.4	456 - 463	0	57	K.LFLPIAIR.L
942.6553	941.6480	941.6062	44.4	456 - 463	0	---	K.LFLPIAIR.L
954.5947	953.5874	953.5447	44.8	376 - 383	0	---	K.IVGIYPHR.K
954.5947	953.5875	953.5447	44.9	376 - 383	0	33	K.IVGIYPHR.K
973.5596	972.5523	972.5240	29.1	99 - 106	1	---	K.KDDVEVLR.R
1037.5256	1036.5183	1036.4825	34.6	246 - 254	0	---	K.EFAEVAESR.T
1187.6296	1186.6223	1186.5731	41.5	350 - 359	0	---	K.NIDGLWENAR.S
1187.6296	1186.6224	1186.5731	41.6	350 - 359	0	81	K.NIDGLWENAR.S
1305.7299	1304.7226	1304.6976	19.2	183 - 194	0	---	R.AGNEYLGLDLLK.E
1306.6624	1305.6551	1305.6598	-3.59	195 - 205	1	---	K.EELIKMTAEAR.Q
1361.8214	1360.8141	1360.7602	39.6	338 - 349	0	---	K.YIDQGLEGIILK.N
1361.8214	1360.8141	1360.7602	39.6	338 - 349	0	85	K.YIDQGLEGIILK.N
1372.7084	1371.7011	1371.6492	37.8	425 - 435	0	---	R.IMENQNYYIGK.I
1391.7972	1390.7899	1390.7357	39.0	152 - 164	1	---	K.FPAFAQLKADGAR.
1708.8512	1707.8439	1707.8138	17.6	385 - 400	1	---	K.DPTKAGGFILESEC
1811.0703	1810.0630	1809.9836	43.9	317 - 332	0	---	K.VILIENQVVNNLDE
1866.0316	1865.0243	1865.0411	-8.98	368 - 383	1	---	K.EVIDVDLKIVGIYP
1880.9402	1879.9329	1879.8954	20.0	108 - 124	1	---	R.VMMRDLECGASVSI
1897.9493	1896.9420	1896.8530	46.9	471 - 487	0	---	K.ANTFEDVFGDFHEV
1897.9493	1896.9421	1896.8530	46.9	471 - 487	0	96	K.ANTFEDVFGDFHEV
1918.1290	1917.1217	1917.0458	39.6	183 - 199	1	---	R.AGNEYLGLDLLKEE
1970.9764	1969.9691	1969.8945	37.9	227 - 243	0	---	K.EPEGLDFLFDAYPE
1994.0941	1993.0868	1993.0368	25.1	81 - 99	1	---	K.LTGNAAIEELTGYI
2099.0916	2098.0843	2097.9895	45.2	226 - 243	1	94	K.KEPEGLDFLFDAYP
2099.0916	2098.0843	2097.9895	45.2	226 - 243	1	---	K.KEPEGLDFLFDAYP
2342.2893	2341.2820	2341.2147	28.8	279 - 297	0	---	K.FQVWDYVPLVEIYS
2342.2893	2341.2820	2341.2147	28.8	279 - 297	0	115	K.FQVWDYVPLVEIYS

No match to: 842.5427, 878.4707, 933.5529, 941.6182, 959.5046, 964.6144, 980.6015, 984.6603, 1016.6838, 1079.5443, 1141.6083, 1151.8319, 1169.5887, 1170.5852, 1189.6296, 1199.6240, 1201.6307, 1219.6141, 1229.6312, 1246.7233, 1261.6564, 1268.6337, 1307.6271, 1338.6040, 1399.7693, 1794.8749, 1887.0306, 1935.8798, 1939.9509, 2189.1868, 2211.2136, 2284.3113, 2285.3208, 2286.2971, 2286.2971, 2289.2871, 2298.3105, 2303.3372, 2303.3372, 2326.2036, 2380.3008, 2417.3369, 2807.4292

2. P19088 **Mass:** 55574 **Score:** 556 **Expect:** 1e-050 **Matches:** 27

DNA ligase OS=Enterobacteria phage T6 GN=30 PE=3 SV=1

Observed	Mr(expt)	Mr(calc)	ppm	Start - End	Miss	Ions	Peptide
845.4611	844.4538	844.4290	29.3	100 - 106	0	---	K.DDVEVLR.R
884.5102	883.5029	883.4803	25.6	42 - 48	0	---	R.GLQYYIK.K
918.4479	917.4406	917.4090	34.4	171 - 178	0	---	R.GDELDDVR.L
921.5546	920.5473	920.5120	38.4	152 - 159	0	---	K.FPAFAQLK.A
921.5546	920.5474	920.5120	38.4	152 - 159	0	47	K.FPAFAQLK.A
942.6553	941.6480	941.6062	44.4	456 - 463	0	57	K.LFLPIAIR.L
942.6553	941.6480	941.6062	44.4	456 - 463	0	---	K.LFLPIAIR.L
954.5947	953.5874	953.5447	44.8	376 - 383	0	---	K.IVGIYPHR.K
954.5947	953.5875	953.5447	44.9	376 - 383	0	33	K.IVGIYPHR.K
973.5596	972.5523	972.5240	29.1	99 - 106	1	---	K.KDDVEVLR.R

1187.6296	1186.6223	1186.5731	41.5	350 - 359	0	---	K.NIDGLWENAR.S
1187.6296	1186.6224	1186.5731	41.6	350 - 359	0	81	K.NIDGLWENAR.S
1305.7299	1304.7226	1304.6976	19.2	183 - 194	0	---	R.AGNEYLGLDLLK.E
1306.6624	1305.6551	1305.6598	-3.59	195 - 205	1	---	K.EELIKMTAEAR.Q
1361.8214	1360.8141	1360.7602	39.6	338 - 349	0	---	K.YIDQGLEGIILK.N
1361.8214	1360.8141	1360.7602	39.6	338 - 349	0	85	K.YIDQGLEGIILK.N
1372.7084	1371.7011	1371.6492	37.8	425 - 435	0	---	R.IMENQNYYIGK.I
1391.7972	1390.7899	1390.7357	39.0	152 - 164	1	---	K.FPAFAQLKADGAR.
1708.8512	1707.8439	1707.8138	17.6	385 - 400	1	---	K.DPTKAGGFILESEC
1811.0703	1810.0630	1809.9836	43.9	317 - 332	0	---	K.VILIENQVVNNLDE
1866.0316	1865.0243	1865.0411	-8.98	368 - 383	1	---	K.EVIDVDLKIVGIYP
1880.9402	1879.9329	1879.8954	20.0	108 - 124	1	---	R.VMMRDLECGASVSI
1897.9493	1896.9420	1896.8530	46.9	471 - 487	0	---	K.ANTFEDVFGDFHEV
1897.9493	1896.9421	1896.8530	46.9	471 - 487	0	96	K.ANTFEDVFGDFHEV
1918.1290	1917.1217	1917.0458	39.6	183 - 199	1	---	R.AGNEYLGLDLLKEE
1994.0941	1993.0868	1993.0368	25.1	81 - 99	1	---	K.LTGNAAIEELTGYI
2298.3105	2297.3032	2297.1885	49.9	279 - 297	0	---	K.FQVWDYVPLVEVYG

No match to: 842.5427, 878.4707, 933.5529, 941.6182, 959.5046, 964.6144, 980.6015, 984.6603, 1016.6838, 1037.5256, 1079.5443, 1141.6083, 1151.8319, 1169.5887, 1170.5852, 1189.6296, 1199.6240, 1201.6307, 1219.6141, 1229.6312, 1246.7233, 1261.6564, 1268.6337, 1307.6271, 1338.6040, 1399.7693, 1794.8749, 1887.0306, 1935.8798, 1939.9509, 1970.9764, 2099.0916, 2099.0916, 2189.1868, 2211.2136, 2284.3113, 2285.3208, 2286.2971, 2286.2971, 2289.2871, 2303.3372, 2303.3372, 2326.2036, 2342.2893, 2342.2893, 2380.3008, 2417.3369, 2807.4292

3. P28376 **Mass:** 52059 **Score:** 61 **Expect:** 0.3 **Matches:** 10

Ribulose bisphosphate carboxylase large chain (Fragment) OS=Acer saccharum GN=

Observed	Mr(expt)	Mr(calc)	ppm	Start End	Miss	Ions	Peptide
1037.5256	1036.5183	1036.4825	34.5	341 - 348	1	---	R.DDFIEKDR.S
1170.5852	1169.5779	1169.5512	22.8	294 - 302	1	---	R.QKNHGMHFR.V +
1187.6296	1186.6223	1186.6571	-29.28	276 - 285	0	---	R.DNGLLLHIHR.A
1187.6296	1186.6224	1186.6571	-29.25	276 - 285	0	11	R.DNGLLLHIHR.A
1261.6564	1260.6491	1260.7078	-46.54	330 - 340	0	---	R.DITLGFVDLLR.D
1708.8512	1707.8439	1707.8693	-14.85	137 - 151	1	---	K.TFQGPPHGIQVER
1794.8749	1793.8676	1793.8076	33.4	227 - 242	0	---	K.GHYLNATAGTCEE
1935.8798	1934.8725	1934.9196	-24.33	437 - 453	1	---	R.EASKWSAELAAAC
2189.1868	2188.1795	2188.0874	42.1	208 - 226	1	---	R.FLFCAEAIFKSQA
2380.3008	2379.2935	2379.1998	39.4	12 - 31	1	---	K.LTYYTPEYVTKDT

No match to: 842.5427, 845.4611, 878.4707, 884.5102, 918.4479, 921.5546, 921.5546, 933.5529, 941.6182, 942.6553, 942.6553, 954.5947, 954.5947, 959.5046, 964.6144, 973.5596, 980.6015, 984.6603, 1016.6838, 1079.5443, 1141.6083, 1151.8319, 1169.5887, 1189.6296, 1199.6240, 1201.6307, 1219.6141, 1229.6312, 1246.7233, 1268.6337, 1305.7299, 1306.6624, 1307.6271, 1338.6040, 1361.8214, 1361.8214, 1372.7084, 1391.7972, 1399.7693, 1811.0703, 1866.0316, 1880.9402, 1887.0306, 1897.9493, 1897.9493, 1918.1290, 1939.9509, 1970.9764, 1994.0941, 2099.0916, 2099.0916, 2211.2136, 2284.3113, 2285.3208, 2286.2971, 2286.2971, 2289.2871, 2298.3105, 2303.3372, 2303.3372, 2326.2036, 2342.2893, 2342.2893, 2417.3369, 2807.4292

Search Parameters

Type of search : **Sequence Query**
Enzyme : **Trypsin**

Fixed modifications : Carbamidomethyl (C)
Variable modifications : Oxidation (M)
Mass values : Monoisotopic
Protein Mass : Unrestricted
Peptide Mass Tolerance : ± 50 ppm
Fragment Mass Tolerance: ± 0.25 Da
Max Missed Cleavages : 1
Instrument type : MALDI-TOF-TOF
Query1 (842.5427,1+) : <no title>
Query2 (845.4611,1+) : <no title>
Query3 (878.4707,1+) : <no title>
Query4 (884.5102,1+) : <no title>
Query5 (918.4479,1+) : <no title>
Query6 (921.5546,1+) : <no title>
Query7 (921.5546,1+) : Locus:1..3.0.9
Query8 (933.5529,1+) : <no title>
Query9 (941.6182,1+) : <no title>
Query10 (942.6553,1+) : Locus:1..3.0.3
Query11 (942.6553,1+) : <no title>
Query12 (954.5947,1+) : <no title>
Query13 (954.5947,1+) : Locus:1..3.0.4
Query14 (959.5046,1+) : <no title>
Query15 (964.6144,1+) : <no title>
Query16 (973.5596,1+) : <no title>
Query17 (980.6015,1+) : <no title>
Query18 (984.6603,1+) : <no title>
Query19 (1016.6838,1+) : <no title>
Query20 (1037.5256,1+) : <no title>
Query21 (1079.5443,1+) : <no title>
Query22 (1141.6083,1+) : <no title>
Query23 (1151.8319,1+) : <no title>
Query24 (1169.5887,1+) : <no title>
Query25 (1170.5852,1+) : <no title>
Query26 (1187.6296,1+) : <no title>
Query27 (1187.6296,1+) : Locus:1..3.0.1
Query28 (1189.6296,1+) : <no title>
Query29 (1199.6240,1+) : <no title>
Query30 (1201.6307,1+) : <no title>
Query31 (1219.6141,1+) : <no title>
Query32 (1229.6312,1+) : <no title>
Query33 (1246.7233,1+) : <no title>
Query34 (1261.6564,1+) : <no title>
Query35 (1268.6337,1+) : <no title>
Query36 (1305.7299,1+) : <no title>
Query37 (1306.6624,1+) : <no title>
Query38 (1307.6271,1+) : <no title>
Query39 (1338.6040,1+) : <no title>
Query40 (1361.8214,1+) : <no title>
Query41 (1361.8214,1+) : Locus:1..3.0.7
Query42 (1372.7084,1+) : <no title>
Query43 (1391.7972,1+) : <no title>
Query44 (1399.7693,1+) : <no title>
Query45 (1708.8512,1+) : <no title>
Query46 (1794.8749,1+) : <no title>
Query47 (1811.0703,1+) : <no title>
Query48 (1866.0316,1+) : <no title>
Query49 (1880.9402,1+) : <no title>
Query50 (1887.0306,1+) : <no title>
Query51 (1897.9493,1+) : <no title>
Query52 (1897.9493,1+) : Locus:1..3.0.5

```
Query53 (1918.1290,1+) : <no title>
Query54 (1935.8798,1+) : <no title>
Query55 (1939.9509,1+) : <no title>
Query56 (1970.9764,1+) : <no title>
Query57 (1994.0941,1+) : <no title>
Query58 (2099.0916,1+) : Locus:1..3.0.10
Query59 (2099.0916,1+) : <no title>
Query60 (2189.1868,1+) : <no title>
Query61 (2211.2136,1+) : <no title>
Query62 (2284.3113,1+) : <no title>
Query63 (2285.3208,1+) : <no title>
Query64 (2286.2971,1+) : <no title>
Query65 (2286.2971,1+) : Locus:1..3.0.8
Query66 (2289.2871,1+) : <no title>
Query67 (2298.3105,1+) : <no title>
Query68 (2303.3372,1+) : Locus:1..3.0.6
Query69 (2303.3372,1+) : <no title>
Query70 (2326.2036,1+) : <no title>
Query71 (2342.2893,1+) : <no title>
Query72 (2342.2893,1+) : Locus:1..3.0.2
Query73 (2380.3008,1+) : <no title>
Query74 (2417.3369,1+) : <no title>
Query75 (2807.4292,1+) : <no title>
```

Mascot: http://www.matrixscience.com/
