# Supplementary material for: Detection of Ligation Products of DNA Linkers with 5′-OH Ends by Denaturing PAGE Silver Stain
Source: PLoS One. 2012 Jun 27;7(6):e39251. doi: 10.1371/journal.pone.0039251 (PMC3384673; doi:10.1371/journal.pone.0039251)
Supplement: Supporting Information S3 — A protocol for MS analysis of T4 DNA ligase. This protocol describes the procedure of MS analysis of T4 DNA ligase (Fermentas). (DOC) [file pone.0039251.s003.doc]

**Supporting information S3**

**Procedures of MS analysis for T4 DNA ligase used**

MS analysis for T4 DNA ligase was performed as described below: 5 µl of T4 DNA ligase (Fermentas, 5U/μl) was reconstituted with 100 µl of 50 mM NH_4_HCO_3_ (pH 8.0). The sample was reduced by adding 10µl of 200 mM DTT / 50 mM NH_4_HCO_3_ (pH 8.0), at 56 ^o^C for 1 hour, and then alkylated by adding 25µl of 200 mM Iodoacetamide / 50 mM NH_4_HCO_3_ (pH 8.0). The mixture was incubated for 1 hour at room temperature in dark. In order for the trypsin to work, 865µl of 50 mM NH_4_HCO_3_ (pH 8.0) were added to make the detergents and other chaotropes to be a compatible final concentration. Then added trypsin solution to a final ratio of 1:30 (w/w, trypsin: protein). Mixed gently by vortex and incubated at 37 ^o^C for 16-20 hrs. Sample was desalted and concentrated by using Millipore ZipTip (µ-C18). The MALDI TOF/TOF samples were prepared by mixing one portion of protein digest sample with one portion of 5 mg/ml α-CHCA solution before spotting on the MALDI plate. MS and tandem mass spectrometry (MS/MS) data were acquired with the AB SCIE x 5800 MALDI TOF/TOF. For MS/MS spectra, the collision energy was 2 keV and CID off. The interpretation of both the MS and MS/MS data was carried out using the ProteinPilot software (AB SCIEX).
